# Adverse childhood experiences, and violence and criminal justice outcomes in adulthood—the moderating role of protective and compensatory childhood experiences

**DOI:** 10.1186/s12916-025-04459-3

**Published:** 2025-11-14

**Authors:** Zara Quigg, Mark A. Bellis, Nadia Butler, Charley Wilson, Jane Harris, Evelyn Hearne, Matthew Millings

**Affiliations:** 1 School of Public and Allied Health, Liverpool John Moores University, Tithebarn Building, Liverpool, L3 2ER UK; 2 Public Health Institute, WHO Collaborating Centre for Violence Prevention, Liverpool John Moores University, Liverpool, UK; 3 School of Justice Studies, Liverpool John Moores University, Liverpool, UK

**Keywords:** Adversity, Resiliency, Childhood, Violence, Incarceration

## Abstract

**Background:**

Globally, there is substantial evidence on the association between adverse childhood experiences (ACEs) and violence and criminal justice exposure. However, emerging research suggests that protective and compensatory childhood experiences (PACEs) may moderate these associations. This study aims to examine the contributing role of PACEs in mitigating the association between ACEs, and violence victimisation and criminal justice exposure in adulthood.

**Methods:**

A cross-sectional representative household survey of adults in a region of the UK examined exposure to ACEs and violence victimisation in adulthood and criminal justice exposure (arrested/incarcerated). Three PACE measures were included: trusted adult, trusted friend, and engagement in extra-curricular activities (e.g. sports teams). Analyses used chi-squared and binary logistic regression.

**Results:**

The odds of experiencing violence or being arrested/incarcerated were higher as ACE count increased, both with and without a trusted adult or friend; however, odds were higher in those without a trusted adult or friend compared to those with a trusted adult or friend across nearly all ACE count categories (e.g. adjusted prevalence of violence victimisation amongst those with 4 + ACEs and no trusted adult, 72% vs. 4 + ACEs with a trusted adult, 53%). The odds of being arrested/incarcerated were also higher in those without engagement in extra-curricular activities compared to those with engagement in extra-curricular activities across nearly all ACE count categories. However, the odds of experiencing violence were higher in those with experience of engaging in extra-curricular activities compared to those without experience of engagement in extra-curricular activities across all ACE count categories.

**Conclusions:**

ACEs, particularly when experienced in accumulation, are associated with increased exposure to violence in adulthood and the criminal justice system. However, associations are moderated by exposure to PACEs, particularly always having a trusted adult or friend in childhood. Surprisingly, the role of extra-curricular activities differed from that of a trusted adult or friend, which warrants further investigation. Focusing prevention efforts on preventing ACEs and building resiliency in childhood is vital for reducing the long-term impacts of ACEs on individuals, communities, and public health and law enforcement services.

## Background

Internationally, there is now strong evidence demonstrating the extent and nature of adverse childhood experiences (ACEs), and their role in increasing risks of harm to individuals, families and communities [1, 2]. ACEs include all forms of child maltreatment (physical, sexual and psychological abuse, and neglect) and growing up in a household or community suffering from significant stressors, such as domestic abuse, substance use, mental ill-health and community violence [1]. A plethora of studies show that the experience of ACEs, particularly when in accumulation, is associated with harm across the lifecourse. Thus, exposure to ACEs is associated with increased risks of ill-health (e.g. physical and mental health; chronic diseases) and engagement in health risk behaviours (e.g. excessive alcohol use; illicit drug use), and other risks such as low educational achievement, and increased risks of poverty, unemployment and homelessness [3–5]. Further, research consistently suggests that exposure to ACEs can increase risks of violence in adolescence and adulthood (as a victim, witness, and/or perpetrator) [3, 6], and emerging evidence suggests increased risk of exposure to the youth and/or criminal justice system [7, 8]. These broad-ranging impacts of ACEs place substantial impacts on society and the attributed costs of ACEs across the lifecourse are substantial, with estimates of US$581 billion in Europe and $748 billion in North America [2].

Findings from a meta-analysis examining associations between ACEs and health and well-being in adulthood demonstrate that compared to those experiencing no ACEs, those experiencing four or more were over seven times more likely to be a victim or perpetrator of violence in adulthood [3]. Further, they are also more likely to engage in health risk behaviours that increase the risk of criminal behaviours. Thus, they are 10 times more likely to report problematic drug use, and over five times more likely to report problematic alcohol use and illicit drug use [3]. A systematic review and meta-analysis of ACEs and justice system contact suggested that there is consistent evidence of a graded association between the number of ACEs experienced (i.e. ACE score) and exposure to the justice system in the United States (US) [7]. However, as most studies focused on exposure to the justice system during youth and young adulthood, they recommend that further research is required examining the association between ACE score and exposure to the justice system in adulthood and later life [7]. A more recent longitudinal US study suggests that the association between ACE score (particularly 4 + ACEs) and exposure to the justice system extends into young and middle adulthood [8], whilst a study of adults in England suggests that compared to those experiencing no ACEs, those experiencing four or more are over 11 times more likely to have ever been incarcerated [9]. Similarly, studies show that those within the youth or criminal justice system have much higher levels of ACEs compared to the general population (e.g. prisons) [10]. For example, in a study of male adults incarcerated in a Welsh prison, nearly all (84.2%) had suffered at least one ACE and 45.5% four or more [10]. A growing body of research suggests that ACEs are associated with increased exposure to violence and/or the criminal justice system because of impacts placed on brain development, emotional regulation and resiliency building (e.g. coping mechanisms) [11, 12].

Critically, experience of ACEs is not deterministic of poor outcomes—not all children who experience ACEs will go on to experience associated harms in adulthood, including exposure to violence or the criminal justice system. Increasingly, emerging evidence suggests that building resiliency in children, families, and communities can help protect or mitigate the impacts of ACEs across the lifecourse [1]. Resilience can be built through raising the hopes and aspirations in children and building skills in self-regulation and executive functioning; ensuring children (and adults) have trusted and supportive relationships; enhancing community networks; and ensuring children, young people and wider community members have access to social and cultural activities [1]. Most research exploring the moderating effect of resilience on associations between ACEs and outcomes has focused on health (e.g. mental illness) or health risk behaviours (e.g. substance use) [13, 14]. Few studies have examined the moderating effect of resilience on outcomes relating to violence or justice exposure (e.g. arrested; incarcerated). A recent longitudinal study in the United Kingdom (UK) examined the association between ACEs and violent outcomes in adolescence and the compensatory role of positive childhood experiences. Findings demonstrate that whilst there are strong associations between having a higher number of ACEs and increased risk of engaging in violence, the risk is reduced when children also had a high number of positive childhood experiences, such as positive peer experiences, participation in activities and hobbies and positive teacher–child relationships [15]. Strengthening the evidence on factors that can protect people from further harm is crucial for taking a more strengths-based approach to mitigating the impacts of ACEs across the lifecourse [16].

This study uses a regional study on ACEs and adulthood violence victimisation to examine the contributing role of sources of childhood resiliency (referred to here as protective and compensatory experiences [PACEs]) in mitigating the association between ACEs, and violence victimisation in adulthood and criminal justice exposure. The study aims to address the following questions:

1. What is the extent of exposure to ACEs, and violence and criminal justice exposure in a sample of adults from a UK region?
2. What is the association between exposure to ACEs, and violence victimisation in adulthood and criminal justice exposure?
3. Do childhood resiliency assets (PACEs) show protective associations with violence victimisation in adulthood and criminal justice exposure, accounting for exposure to ACEs?

We hypothesise that (i) experience of ACEs has a dose–response relationship with violence and criminal justice exposure, with risks increasing as ACE count increases; and (ii) PACEs moderate this relationship across ACE count, reducing risks of violence and criminal justice exposure.

## Methods

### Design and sampling

A cross-sectional survey of adults (aged 18 + years) resident in households in an English region (population 1.16 million adults; [17]) was implemented from November 2023 to April 2024. The survey was conducted collaboratively by Liverpool John Moores University and a regional Violence Reduction Partnership, a public sector body tasked by the UK Government to implement a public health approach to violence prevention. The survey aimed to understand perceptions and experiences of community safety, and the nature and extent of violence, including ACEs, to inform prevention activity. The study utilised quota sampling to select 110 Lower Super Output Areas (LSOAs; small geographic areas with a population of around 1500 residents) stratified by English Index of Multiple Deprivation (IMD) quintile, age group, and sex, from the five local authority areas in the study region. Ethical approval for the study was granted by Liverpool John Moores Research Ethics Committee (23/PHI/050). No incentives were offered or provided to study participants.

### Study recruitment

Within each selected LSOA, up to 500 randomly selected households were sent a postal letter describing the study, and its voluntary and confidential nature. The letter contacted households with the option to take part in the survey online or opt-out of the study. If a member of the household did not complete the survey online, and had not opted out of the study, the letter informed the household that a trained interviewer from a market research company may visit their household to invite them to take part in the survey in-person within the next 5 weeks. Households were visited across all days of the week, at varying times from 9 am to 9 pm (if there was no answer, interviewers visited again up to five times until there was an answer). If an individual was ineligible (e.g. aged under 18 years; not a resident of the household) or declined to participate in the study, the interviewer recorded the outcome of the contact then moved on to the next randomly selected household. Only one adult per household was eligible to participate in the study. If more than one individual in a household was eligible and available, the interviewers would ask for the person whose birthday is next to take part. The study utilised computer-assisted personal interviewing technology, with computer-assisted self-interviewing used for more sensitive parts of the survey (e.g. questions on experience of ACEs and violence victimisation).

### Response rate

A total of 54,761 postal letters were distributed to the randomly selected households in the randomly selected LSOAs. At this stage, 1215 participants completed the survey online, and 467 households opted out of the study. Thus, at this stage, 2.2% of households receiving a letter (who had not opted out) completed the survey. Subsequently, 6040 household visits were made by an interviewer where an eligible participant answered the door, and of these 4180 completed the survey in person (thus the response rate amongst households visited by a field researcher is 69.2%; comparable to other UK household surveys on ACEs [18]). Overall, 5395 participants completed the survey. This sample size was selected as 500 individuals with four or more ACEs were needed to meet the wider aims of the project, and other studies suggested that this sample size would be adequate for this.

### Measures

#### Adverse childhood experiences (ACEs)

The survey included nine ACEs commonly examined in ACE household surveys [1, 9] and based on the original ACE study by Felitti et al. [19], including whether the individual before the age of 18 years experienced physical, verbal, or sexual abuse; and household stressors including if their parents had separated/divorced, if they had witnessed domestic violence, and if they lived with anyone who had problems with alcohol or drugs, was mentally ill, or had been incarcerated. Response options included yes, no, or prefer not to say. Like existing research on ACEs, the number of ACEs was summed to provide a total ACE count (0 ACEs, 1 ACE, 2–3 ACEs, 4 + ACEs). Across the nine ACE questions, the proportion with missing data or a ‘preferred not to say’ response ranged from 4.8 to 7.7%; only those who selected yes or no to at least four ACE questions (our highest ACE count category) were included in our analyses.

#### Protective and compensatory childhood experiences (PACEs)

Participants were asked if, while they were growing up before the age of 18, there was an adult in their life who they could trust and talk to about any personal problems, and if they had friends in their life who they could trust and talk to about any personal problems. Response options included never, sometimes, always, and prefer not to say. Participants were also asked while they were growing up, before the age of 18, if they were engaged in any extra-curricular or community activities (e.g. sports clubs/teams; dance, drama, or arts clubs; cubs, brownies, scouts, guides; volunteering; etc.). Response options included yes, no, and prefer not to say. Responses for each PACE were grouped into always having a trusted adult (yes/no; trusted adult), always having a trusted friend (yes/no; trusted friend), and having ever engaged in any extra-curricular or community activity (yes/no; activities). Across the three PACE questions, the proportion with missing data or a ‘preferred not to say’ response ranged from 3.2 to 7.8%; only those who selected yes or no were included in our analyses.

#### Violence victimisation

Violence victimisation was measured using seven items and included whether, after the age of 18 years, the individual experienced: physical violence; psychological abuse and coercive control; stalking and harassment; indecent exposure; unwanted sexual touching; and rape or assault by penetration. Response options included yes, no, and prefer not to say (for analyses, cases selecting prefer not to say across all questions were excluded). Those experiencing violence were asked a follow-up question to identify if the violence had occurred in the past 12 months. For analyses, two outcome variables were produced: any type of violence victimisation since age 18 years (violence ever) and any type of violence victimisation in the past 12 months (violence past year).

#### Criminal justice exposure

Criminal justice exposure was measured through two questions: ‘Have you ever been arrested in the UK?’ (arrested ever) and ‘Have you ever spent a night in prison or jail in the UK?’ (incarcerated ever). Response options included yes, no, and prefer not to say (only those who selected yes or no were included in our analyses).

#### Co-variates

Socio-demographics examined in bi-variate analyses and controlled for in regression models included age group (18–24, 25–54, 55 + years), sex (male, female), ethnicity (any white ethnicity; any non-white ethnicity) and deprivation quintile (1 least deprived–5 most deprived; based on the English IMD). IMD is an area-level measure used in the UK to assess the relative deprivation of an area (here lower super output areas) compared to another and is based on categories including income, employment, health, education, access to services, community safety and physical environment.

### Analyses

All analyses were conducted using SPSS version 28. Bivariate analyses using chi-squared explored associations between violence and criminal justice outcomes, and all covariates, ACE count and each individual PACE. Generalised linear models using binary logistic regression (enter method) were used to examine the independent associations between ACE count and violence (ever/past year) and criminal justice outcomes (arrested/incarcerated), while controlling for sociodemographics. 

To examine the moderating role of PACEs, ACE count and each individual PACE were included as a single variable categorised into all possible combinations of ACE count and PACE (e.g. 4 + ACEs and no trusted adult). A subsequent suite of generalised linear models using binary logistic regression (enter method) was used to examine the independent associations between each individual PACE and ACE count (combined), and violence and criminal justice outcomes, while controlling for sociodemographics. Here, due to low numbers, only violence ever and ever being arrested or incarcerated (combined) were examined as outcomes. Estimated marginal means were used to estimate the adjusted prevalence of each outcome for groups of each ACE count and individual PACE.

## Results

### ACEs, and violence and criminal justice exposure

Across the sample, 51.6% reported at least one ACE, and 15.9% reported 4 + ACEs (19.2% 1 ACE, 16.4% 2–3). A third (34.5%) reported having ever experienced violence victimisation in adulthood (4.4% past year), and 8.7% had ever been arrested and 5.2% spent a night in a UK prison/jail. The proportion reporting each outcome increased as ACE count increased (*p* < 0.001) (Table 1). Women reported significantly higher levels of violence victimisation ever than men (women 36.0%, men 32.7%; *p* < 0.05), whilst men reported significantly higher levels of ever being arrested or incarcerated than women (arrested: women 3.5%, men 14.6%; *p* < 0.001 / incarcerated: women 1.8%, men 9.1%; *p* < 0.001). Significant differences were found for each outcome by age group, and those of any white ethnicity reported significantly higher levels of all outcomes (except for past year violence). There were significant differences by deprivation for all outcomes (except violence ever), with the prevalence of each outcome tending to be higher in areas of highest deprivation.

**Table 1:** Bivariate relationships between violence and criminal justice outcomes, and ACE count, individual PACEs and socio-demographics

			Violence (ever)	Violence (past year)	Arrested (ever)	Incarcerated (ever)
ACE count		No ACEs	19.1%	1.8%	4.5%	2.3%
		1 ACE	36.1%	3.6%	8.2%	5.0%
		2–3 ACEs	49.5%	5.7%	12.9%	7.8%
		4 + ACEs	63.9%	12.1%	17.4%	11.7%
		*X*^2^	619.4	150	140.1	115.4
		*P*	< 0.001	< 0.001	< 0.001	< 0.001
PACE—always trusted adult		No	55.7%	9.2%	15.4%	11.5%
		Yes	32.9%	4.1%	8.0%	4.7%
		*X*^2^	74.7	19.8	22	30.6
		*P*	< 0.001	< 0.001	< 0.001	< 0.001
PACE—always trusted friend		No	48.6%	6.4%	13.0%	9.7%
		Yes	33.5%	4.3%	8.2%	4.8%
		*X*^2^	29.1	3.2	8.5	14.3
		*P*	< 0.001	0.074	0.003	< 0.001
PACE—ever activities		No	30.1%	3.9%	9.6%	6.3%
		Yes	36.1%	4.6%	8.3%	4.9%
		*X*^2^	14.5	0.9	1.9	3.6
		*P*	< 0.001	0.333	0.167	0.059
Sex		Male	32.7%	3.9%	14.6%	9.1%
		Female	36.0%	4.9%	3.5%	1.8%
		*X*^2^	5.7	2.9	185.7	130
		*P*	0.017	0.091	< 0.001	< 0.001
Age group (years)		18–24	28.1%	11.2%	3.8%	1.7%
		25–54	39.5%	5.6%	10.0%	6.4%
		55 +	30.8%	2.0%	8.3%	4.7%
		*X*^2^	45.4	84.1	18	18.3
		*P*	< 0.001	< 0.001	< 0.001	< 0.001
Any White ethnic background		No	26.2%	7.9%	5.6%	3.3%
		Yes	35.0%	4.1%	8.8%	5.3%
		*X*^2^	9.7	9.5	3.8	2.4
		P	0.002	0.002	0.052	0.125
Deprivation quintile		1 (least deprived)	29.0%	2.7%	5.0%	2.8%
		2	33.0%	1.8%	5.7%	2.8%
		3	34.6%	3.1%	7.4%	4.6%
		4	33.8%	4.7%	7.7%	5.6%
		5 (most deprived)	36.2%	6.0%	11.1%	6.6%
		*X*^2^	8.4	29.7	32.7	22.4
	*P*		0.079	< 0.001	< 0.001	< 0.001

In multi-variate analyses, controlling for age, sex, ethnicity and deprivation, ACE count was significantly associated with all outcomes, with an increase in exposure to outcomes as ACE count increased (Table 2). Thus, compared to those with zero ACEs, the odds of experiencing violence ever were 2.35, 4.07 and 7.30 times higher amongst those experiencing 1, 2–3 and 4 + ACEs respectively (*p* < 0.001) (past year violence adjusted odds ratios: 1 ACE 1.98, 2–3 ACEs 3.09 and 4 + ACEs 6.48; *p* < 0.01). Similarly, compared to those with zero ACEs, the odds of ever being arrested were 1.80, 2.94 and 4.72 times higher, and the odds of ever being incarcerated were 2.12, 3.31 and 5.91 times higher, amongst those experiencing 1, 2–3 and 4 + ACEs respectively.

**Table 2:** Adjusted odds ratios for violence and criminal justice outcomes—ACE count and sociodemographics

		Victim of violence (ever)		Victim of violence (past year)		Arrested (ever)		Incarcerated (ever)	
		AOR	*p*	AOR	*p*	AOR	*p*	AOR	*p*
ACE count	4 + ACEs	7.30	< 0.001	6.48	< 0.001	4.72	< 0.001	5.91	< 0.001
	2–3 ACEs	4.07	< 0.001	3.09	< 0.001	2.94	< 0.001	3.31	< 0.001
	1 ACE	2.35	< 0.001	1.98	0.004	1.80	< 0.001	2.12	< 0.001
	0 ACEs	1.00		1.00		1.00		1.00	
Sex	Female	1.11	0.127	1.10	0.529	0.18	< 0.001	0.15	< 0.001
	Male	1.00		1.00		1.00		1.00	
Age group (years)	55 +	1.29	0.048	0.19	< 0.001	2.53	< 0.001	3.24	0.003
	25–54	1.65	< 0.001	0.44	< 0.001	3.03	< 0.001	4.36	< 0.001
	18–24	1.00		1.00		1.00		1.00	
Any White ethnic background	Yes	1.50	0.005	0.62	0.043	1.74	0.037	1.76	0.096
	No	1.00		1.00		1.00		1.00	
Deprivation quintile	5 (Most)	1.11	0.445	1.34	0.390	2.17	0.003	2.13	0.027
	4	1.08	0.599	1.26	0.540	1.34	0.308	1.69	0.154
	3	1.20	0.222	0.87	0.729	1.43	0.210	1.55	0.238
	2	1.13	0.405	0.62	0.257	1.05	0.874	0.88	0.757
	1 (least)	1.00		1.00		1.00		1.00	

*AOR* adjusted odds ratio

### ACEs, PACEs and violence and criminal justice exposure

Most of the sample reported having each of the childhood resilience assets (PACEs): a trusted adult (always; 92.7%); a trusted friend (always; 93.5%); and engagement in extra-curricular or community activities (ever; 74.4%). In bivariate analyses, there were significant differences in exposure to nearly all outcomes by each individual PACE, with a general decrease in exposure to outcomes with the presence of each PACE (except for extracurricular activity and violence [ever, past year], which increased) (Table 1).

**Fig. 1:**
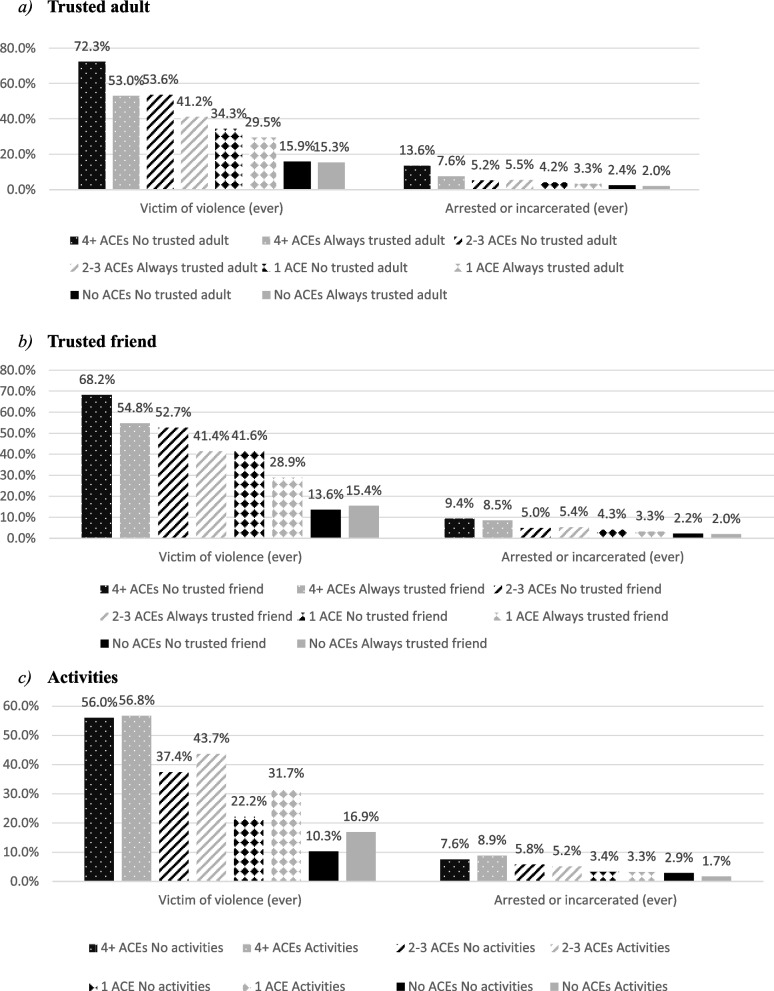
Adjusted extent of violence victimisation and criminal justice exposure by ACE count and PACE (*controlling for sex, age, ethnicity, and deprivation*)

In multi-variate analyses, three models were run, one for each PACE. To examine the moderating role of PACEs, ACE count and each individual PACE were included as a single variable categorised into all possible combinations of ACE count and PACE (e.g. 4 + ACEs and no trusted adult). In model 1 (trusted adult; Table 3), controlling for age, sex, ethnicity and deprivation, the odds of experiencing violence ever, or being arrested or incarcerated were higher as ACE count increased, both with and without a trusted adult; however, odds were higher in those without a trusted adult compared to those with a trusted adult across all ACE count categories. Estimated marginal means shows that in those with 4 + ACEs and no trusted adult, the adjusted prevalence of violence victimisation (ever) was 72%; however this reduced to 53% amongst those with 4 + ACEs who had a trusted adult (Fig. 1). Similarly, the adjusted prevalence of ever being arrested or incarcerated amongst those with 4 + ACEs reduced from 14% when there was no trusted adult present to 8% if there was a trusted adult present during childhood (Fig. 1).

**Table 3:** Adjusted odds ratios for violence and criminal justice outcomes—ACE count and PACE trusted adult, and sociodemographics

		Victim of violence (ever)		Arrested or incarcerated (ever)	
		AOR	*p*	AOR	*p*
ACE count * PACE trusted adult	4 + ACEs and No trusted adult	14.43	< 0.001	7.84	< 0.001
	4 + ACEs and a trusted adult	6.21	< 0.001	4.09	< 0.001
	2–3 ACEs and No trusted adult	6.38	< 0.001	2.72	0.009
	2–3 ACEs and a trusted adult	3.86	< 0.001	2.90	< 0.001
	1 ACE and No trusted adult	2.89	< 0.001	2.18	0.119
	1 ACE and a trusted adult	2.30	< 0.001	1.72	< 0.001
	0 ACEs and No trusted adult	1.04	0.882	1.24	0.634
	0 ACEs and a trusted adult	1.00		1.00	
Sex	Female	1.09	0.197	0.19	0.000
	Male	1.00		1.00	
Age group (years)	55 +	1.24	0.093	2.50	< 0.001
	25–54	1.62	< 0.001	3.03	< 0.001
	18–24	1.00		1.00	
Any White ethnic background	Yes	1.51	0.005	1.82	0.025
	No	1.00		1.00	
Deprivation quintile	5 (Most)	1.09	0.518	1.87	0.011
	4	1.06	0.719	1.11	0.692
	3	1.19	0.249	1.32	0.312
	2	1.12	0.459	0.87	0.611
	1 (least)	1.00		1.00	

*AOR* adjusted odds ratio

In model 2 (trusted friend; Table 4), controlling for age, sex, ethnicity and deprivation, the odds of experiencing violence ever, or being arrested or incarcerated were higher as ACE count increased, both with and without a trusted friend; however, odds were higher in those without a trusted friend compared to those with a trusted friend across nearly all ACE count categories (except for arrest/incarceration, 2–3 ACEs). Estimated marginal means shows that in those with 4 + ACEs and no trusted friend, the adjusted prevalence of violence victimisation (ever) was 68%; however this reduced to 55% amongst those with 4 + ACEs who had a trusted friend (Fig. 1). Adjusted prevalence of ever being arrested or incarcerated was slightly lower across ACE counts when there was a trusted friend present (except for 2–3 ACEs; Fig. 1).

**Table 4:** Adjusted odds ratios for violence and criminal justice outcomes—ACE count and PACE trusted friend, and sociodemographics

		Victim of violence (ever)		Arrested or incarcerated (ever)	
		AOR	*p*	AOR	*p*
ACE count * PACE trusted friend	4 + ACEs and No trusted friend	11.78	< 0.001	5.14	< 0.001
	4 + ACEs and a trusted friend	6.64	< 0.001	4.64	< 0.001
	2–3 ACEs and No trusted friend	6.12	< 0.001	2.61	0.040
	2–3 ACEs and a trusted friend	3.88	< 0.001	2.84	< 0.001
	1 ACE and No trusted friend	3.91	< 0.001	2.21	0.085
	1 ACE and a trusted friend	2.23	< 0.001	1.72	< 0.001
	0 ACEs and No trusted friend	0.86	0.571	1.15	0.725
	0 ACEs and a trusted friend	1.00		1.00	
Sex	Female	1.10	0.140	0.19	< 0.001
	Male	1.00		1.00	
Age group (years)	55 +	1.26	0.074	2.61	< 0.001
	25–54	1.64	< 0.001	3.14	< 0.001
	18–24	1.00		1.00	
Any White ethnic background	Yes	1.51	0.005	1.78	0.031
	No	1.00		1.00	
Deprivation quintile	5 (Most)	1.08	0.564	1.86	0.012
	4	1.05	0.733	1.12	0.668
	3	1.18	0.261	1.30	0.338
	2	1.12	0.461	0.85	0.569
	1 (least)	1.00		1.00	

*AOR* adjusted odds ratio

In model 3 (extracurricular activities; Table 5), controlling for age, sex, ethnicity and deprivation, the odds of experiencing violence ever or being arrested/incarcerated were generally higher as count increased, both with and without activities. However, for violence ever, odds were higher in those with activities compared to those without activities across all ACE count categories. Whilst for arrested/incarcerated, odds were higher in those without activities compared to those with activities across all ACE count categories except for 4 + ACEs.

**Table 5:** Adjusted odds ratios for violence and criminal justice outcomes – ACE count and PACE activities, and sociodemographics

		Victim of violence (ever)		Arrested or incarcerated (ever)	
		AOR	*p*	AOR	*p*
ACE count * PACE activities	4 + ACEs and No activities	6.27	< 0.001	4.78	< 0.001
	4 + ACEs and activities	6.47	< 0.001	5.67	< 0.001
	2–3 ACEs and No activities	2.95	< 0.001	3.62	< 0.001
	2–3 ACEs and activities	3.82	< 0.001	3.20	< 0.001
	1 ACE and No activities	1.40	0.047	2.02	0.015
	1 ACE and activities	2.29	< 0.001	1.96	< 0.001
	0 ACEs and No activities	0.56	< 0.001	1.71	0.009
	0 ACEs and activities	1.00		1.00	
Sex	Female	1.12	0.093	0.19	< 0.001
	Male	1.00		1.00	
Age group (years)	55 +	1.36	0.016	2.61	< 0.001
	25–54	1.69	< 0.001	3.15	< 0.001
	18–24	1.00		1.00	
Any White ethnic background	Yes	1.44	0.013	1.87	0.020
	No	1.00		1.00	
Deprivation quintile	5 (Most)	1.17	0.257	1.90	0.009
	4	1.09	0.546	1.14	0.621
	3	1.21	0.200	1.30	0.333
	2	1.13	0.398	0.89	0.677
	1 (least)	1.00		1.00	

*AOR* adjusted odds ratio

## Discussion

This study aimed to examine the association between ACEs and violence and criminal justice outcomes, and if and how these associations may be moderated by the presence of PACEs. Using data from a representative population survey of adults in a UK region, it demonstrates that a significant proportion of adults have been exposed to adversity in childhood and, or adulthood. Importantly, the study demonstrates a strong graded association between ACE count and violence victimisation in adulthood, as well as having ever been exposed to the UK criminal justice system, with these associations starting at exposure to one or more ACEs. Further, the study demonstrates the critical, and potentially varying role of PACEs in moderating these associations. Thus, the presence of a trusted adult or trusted friend in childhood appears to be protective against poor outcomes in adulthood. However, the role of extra-curricular activities differed from that of a trusted adult or friend.

Our study found that nearly half (51.6%) of the sample reported at least one ACE, and around one in ten (15.9%) reported 4 + ACEs. A third (34.5%) of our sample had experienced violence since the age of 18 years (4.4% in the past year), and over one in twenty had ever been arrested (8.7%) or incarcerated (5.2%) in the UK. The exposure to ACEs in our study is similar to that of other UK and international ACE studies [2], with slight variance in terms of exposure to violence victimisation (in the past year) and the criminal justice system (e.g. Wales’ past year violence victimisation at 9.1% [20]). Critically however, our study strengthens the evidence base that demonstrates how exposure to ACEs is associated with an increased likelihood of exposure to adversity in adulthood [3, 9]. Our study, importantly, shows that whilst there are dose–response associations between ACEs and violence victimisation in adulthood and criminal justice exposure, associations start with exposure to just one ACE. That our study, consistent with other seminal studies in this field [2] captures that a large proportion of the population experiences at least one ACE emphasises how critical a public health issue the prevention of ACEs, as well as violence and criminal justice exposure has become.

Preventing ACEs is now a vital mission across communities, organisations and governments, and our study raises the importance of early intervention to break the lifelong cycle of adversity and violence. This is particularly so as several studies show that the links between ACEs and increased risks of violence and criminal justice contact are evident even during adolescence [7, 8, 21]. ACEs can negatively impact healthy brain development, which can increase the risk of emotional and conduct problems, and risk-taking behaviours in adolescence and adulthood [16, 22]. Further, there is a wealth of evidence demonstrating how ACEs increase the risk of health-harming behaviours (e.g. substance use), poor academic achievement, unemployment and poor mental health which may further increase risks of violence and criminal justice exposure [1]. Within this context and given the prevalence of ACEs globally [2], emerging evidence demonstrates the positive role of building resiliency in childhood and adulthood to mitigate the impacts of ACEs across the lifecourse [13, 15, 23, 24]. For those working to commission and/or administer interventions that seek to stimulate efforts to nurture childhood resilience our study offers crucial insights into the moderating roles PACEs—in the form of trusted adults and friends—can play in managing the legacy impacts of ACEs.

Our study demonstrates the vital role of always having a trusted adult during childhood in reducing risks of violence victimisation and criminal justice exposure [13, 25]. The odds of experiencing violence or being arrested or incarcerated were higher as ACE count increased, both with and without a trusted adult. However, odds were higher in those without a trusted adult compared to those with a trusted adult across all ACE count categories. Thus, investing in programmes that provide opportunities for children to build positive trusting and safe relationships with adults is vital. These may include parenting programmes that aim to build strong parent–child bonds (and prevent ACEs more broadly) [1], and programmes that connect children with trusted adults outside of the family home, such as within schools or other community spaces [26]. Further research is needed in this area to explore whether sequential or concurrent relationships with associated adults—such as social workers, teachers, youth workers and extended family members—can play a similar positive compensatory role in children’s lives to a long-term relationship with a single trusted adult [27].

The study also demonstrates the vital role of always having a trusted friend during childhood in reducing risks of violence victimisation and criminal justice exposure. As with a trusted adult, the odds of experiencing violence, or being arrested or incarcerated were higher as ACE count increased, both with and without a trusted friend. However, odds were higher in those without a trusted friend compared to those with a trusted friend across nearly all ACE count categories. International studies suggest trusted friendships can protect against negative mental health, behavioural, and social outcomes amongst youth with ACEs [28–30], but there is limited evidence on violence victimisation and criminal justice exposure [30]. Peer relationships during childhood and adolescence are multi-faceted and their influence is dependent on factors including relationship quality, peer characteristics and peer influence [30]. ACEs often co-occur with disrupted peer relationships which may lead to fewer opportunities to build strong emotional connections [29]. Furthermore, rejection by peers and associating with peers who reinforce aggressive and anti-social behaviours has been linked to violent behaviours in later life [31]. A recent systematic review found the negative wellbeing effects of ACEs were typically mitigated by positive peer characteristics (e.g. prosocial peers) and exacerbated by negative peer characteristics (e.g. substance use, engagement in antisocial behaviour) [30]. The wider research evidence recommends that when intervention activities with young people take place these should aim to facilitate positive peer relationships for young people through prosocial activities and environments [31, 32].

Extracurricular activity participation has the capacity to expand young people’s social networks with non-familial adults and peers [32]. A recent UK study, for example, found regular sports participation significantly reduced the negative psychological consequences of ACEs [33]. However, whilst our study found that ever engaging in extracurricular activities significantly decreased the risk of exposure to the criminal justice system (across nearly all ACE count categories), we found that it increased the odds of ever experiencing violence. These findings are, in part, consistent with previous longitudinal studies that similarly report mixed impacts of extracurricular activities on externalising behaviours (including violence and aggression) depending on the extracurricular activity, peer affiliations formed, and outcome studied [31, 32, 34]. This suggests that context is important when considering the role of extracurricular activities in enhancing childhood resilience including who engages in these activities, where they are located, and the dynamics of what participation involves. A recent UK survey of 10,000 young people found that those who have been affected by violence are twice as likely to go to a youth club (60% who had been victims of violence, and 65% of those who have perpetrated violence) compared to those who haven’t been victims or perpetrators of violence (31%) [35]. This suggests youth services and other extracurricular organisations are proactively recruiting and supporting young people living in areas where they are at greater risk of exposure to crime, anti-social behaviour and violence. This could partially contribute to the heightened levels of violent victimisation among those who had ever engaged with extracurricular activities in our study. In addition, research suggests young people living in areas of higher social deprivation generally engage in extracurricular activities less frequently and are less likely to sustain these activities into middle and late adolescence due to resources, logistics, and competing demands. This may reduce their opportunities to develop longer term positive connections with peers and, in particular, trusted supervising adults which our study found reduced the risk of violence victimisation [32]. Future intervention programmes may be successful if they aim to reduce the physical and financial barriers to extracurricular participation and train supervising staff to create a positive social environment that manages participant vulnerability and reduces the risk of peer rejection and negative peer dynamics [31, 32].

### Limitations and future research

Whilst our study has similar ACE counts to other studies [2], data on ACEs were collected retrospectively via self-report and thus may be affected by recall bias or an unwillingness to report (this also means that the prevalence of ACEs should be viewed as a minimum count). Further, data were collected from one UK region, and therefore findings may not be generalisable to other populations. As this study formed part of a wider study, we were not able to include a validated scale on PACEs and rather used three questions to identify the presence of three PACE types. Whilst similar questions have been used in comparable ACE studies (e.g. [13]), future research should consider using a validated scale to measure PACEs (e.g. [12]). Similar to other studies (e.g. (13)), our study used IMD to control for the socioeconomic area in which participants resided during survey completion (i.e. adulthood). Future studies should consider including a measure of socioeconomic circumstances during childhood, as these may confound the association of ACEs and PACEs with outcomes in adulthood. However, given our findings and discussion around if and how extra-curricular activities may or may not moderate associations between ACEs and violence or criminal justice outcomes across the lifecourse, further research should explore the context, mechanisms and outcomes of interventions involving extra-curricular activities to prevent such exposure, and which types of interventions are likely to be most beneficial in protecting children from future harm.

## Conclusions

There is now a wealth of evidence demonstrating the links between ACEs and violence and criminal justice exposure in adulthood, along with a wealth of other negative outcomes. Our study adds to this evidence base and shows that ACEs, particularly when experienced in accumulation, are associated with increased exposure to violence in adulthood and the criminal justice system. Critically, however, we are able to demonstrate that these associations are prevalent even with exposure to one ACE. Within this context our study adds weight to the growing acknowledgement and emerging evidence on the positive impacts of PACEs in mitigating the impacts of ACEs. This study highlights the importance of building childhood resiliency to better manage the lingering and legacy impacts of ACEs and, in particular, shows the positive impacts of having access to a trusted adult and/or friend. Focusing prevention efforts on preventing ACEs and building PACEs is vital for reducing the long-term impacts of ACEs on individuals, communities, and public health and law enforcement services.

## Data Availability

The dataset analysed in the current study is available from the corresponding author on reasonable request.

## Abbreviations

ACEs: Adverse childhood experiences
LSOAs: Lower Super Output Areas
IMD: English Index of Multiple Deprivation
PACEs: Positive and compensatory experiences
UK: United Kingdom
US: United States
